# Dr. Harlan on Acetate of Lead in Dysentery and Malignant Cholera

**Published:** 1838-06-06

**Authors:** R. Harlan

**Affiliations:** Philadelphia


### Dr. Harlan *on Acetate of Lead in Dysentery and Malignant Cholera.*

Philadelphia, May 28th, 1838.

To the Editors of the Medical Examiner:

*Gentlemen:*—In the American Journal of the Medical Sciences, for May, 1838, there is a detailed account, from the London Medical Gazette, of an article, entitled "On the Treatment of Cholera, by R. J. Graves, M. D," who claims as original, the use of Acetate of Lead in this disease. It has been long a subject of complaint against many editors of American Medical Journals, that, in their anxiety to collate and disseminate the latest professional intelligence, from abroad, they, too frequently, neglect the meritorious claims of their brethren, at home.

In the present instance, it is scarcely to be presumed, that the editor of the American Journal of Medical Sciences is ignorant of the claims of the writer to originality, in the application of sugar of lead to the treatment of Dysentery and Malignant Cholera.

In the American Medical Recorder, published in Philadelphia, in 1821, will be found a memoir, by the writer, entitled, "Cases illustrative of the good effects of sugar of lead in Dysentery." After the detail of cases, successfully treated by the internal use of sugar of lead, the author remarks: "It appears, from the works of Jackson and also of Moseley, that sugar of lead has been used, both in acute and chronic dysentery, in the West Indies; I am convinced, however, that this remedy was not used, in such large doses, with similar views, nor so efficiently, previously to my having used it so very extensively and beneficially, in the obstinate epidemic of 1820. There is no evidence, indeed, that the remedy in question was ever prescribed in acute dysentery, before the summer of 1820, as above referred to."

As a remedy in dysentery, this medicine has since been extensively resorted to by the profession in succeeding epidemics, both in public and private practice. The successful application of the saccharum saturni to the treatment of dysentery, led the author, very naturally, to the trial of this remedy in malignant cholera, when it occurred in our city, in 1832.

The result of the author's experience in this disease, was published in January, 1835; see "Observations on the Malignant Cholera, as it occurred in Philadelphia, in 1832—Harlan's Medical and Physical Researches." Very few of the numerous cases treated in the municipal hospital under the control of the author, were here published. In some of these, however, the sugar of lead, as a valuable adjuvant in this disease, stands prominent as one of the means resorted to in the treatment of this fearful malady.

In the work above quoted, the article on the use of sugar of lead in dysentery was republished.

So much for the claims of the writer to originality in the application of sugar of lead, to the treatment of dysentery and malignant cholera. At the same time, he willingly concedes to Dr. Graves the same merit which he claims himself. According to this author's own statement, he, also, was led, for the first time, to the use of the saturnine remedy in the cholera, as it prevailed in Dublin, in July, 1834, by previously observing its good effects in dysentery and diarrhœa, during the months of May and June of 1834.

The doses of the sugar of lead, used by Dr. Graves, were smaller than those previously recommended by American practitioners.

R. Harlan, M.D.

[The article alluded to, on the treatment of Cholera, by Dr. Graves, appeared also in this Journal, for January 17th, and was copied into other medical periodicals, as well as into the American Journal for May. The omission to notice Dr. Harlan's priority of claim to the recommendation of the remedy in question, was an inadvertence, which we are pleased to have the opportunity to correct, and we presume that the other delinquent Journals will hasten to make a similar *amende.*—Eds.]

